# Efficiency of Immunotoxin Cytotoxicity Is Modulated by the Intracellular Itinerary

**DOI:** 10.1371/journal.pone.0047320

**Published:** 2012-10-09

**Authors:** Lori L. Tortorella, Nina H. Pipalia, Sushmita Mukherjee, Ira Pastan, David Fitzgerald, Frederick R. Maxfield

**Affiliations:** 1 Department of Biochemistry, Weill Cornell Medical College, New York, New York, United States of America; 2 Laboratory of Molecular Biology, National Cancer Institute, Bethesda, Maryland, United States of America; Institute of Molecular and Cell Biology, Singapore

## Abstract

*Pseudomonas* exotoxin-based immunotoxins, including LMB-2 (antiTac(F_v_)-PE38), are proposed to traffic to the trans-Golgi network (TGN) and move by a retrograde pathway to the endoplasmic reticulum, where they undergo translocation to the cytoplasm, a step that is essential for cytotoxicity. The retrograde transport pathways used by LMB-2 are not completely understood, so it is unclear if transit through specific organelles is critical for maximal cytotoxic activity. In this study, we used Chinese hamster ovary (CHO) cell lines that express chimeric constructs of CD25, the Tac antigen, attached to the cytoplasmic domain of the TGN-targeted transmembrane proteins, TGN38 and furin. These chimeras are both targeted to the TGN, but the itineraries they follow are quite different. LMB-2 was incubated with the two cell lines, and the efficiency of cell killing was determined using cell viability and cytotoxicity assays. LMB-2 that is targeted through the endocytic recycling compartment to the TGN via Tac-TGN38 kills the cells more efficiently than immunotoxins delivered through the late endosomes by Tac-furin. Although the processing to the 37 kDa active fragment was more efficient in Tac-furin cells than in Tac-TGN38 cells, this was not associated with enhanced cytotoxicity – presumably because the toxin was also degraded more rapidly in these cells. These data indicate that trafficking through specific organelles is an important factor modulating toxicity by LMB-2.

## Introduction

**Figure 1:**
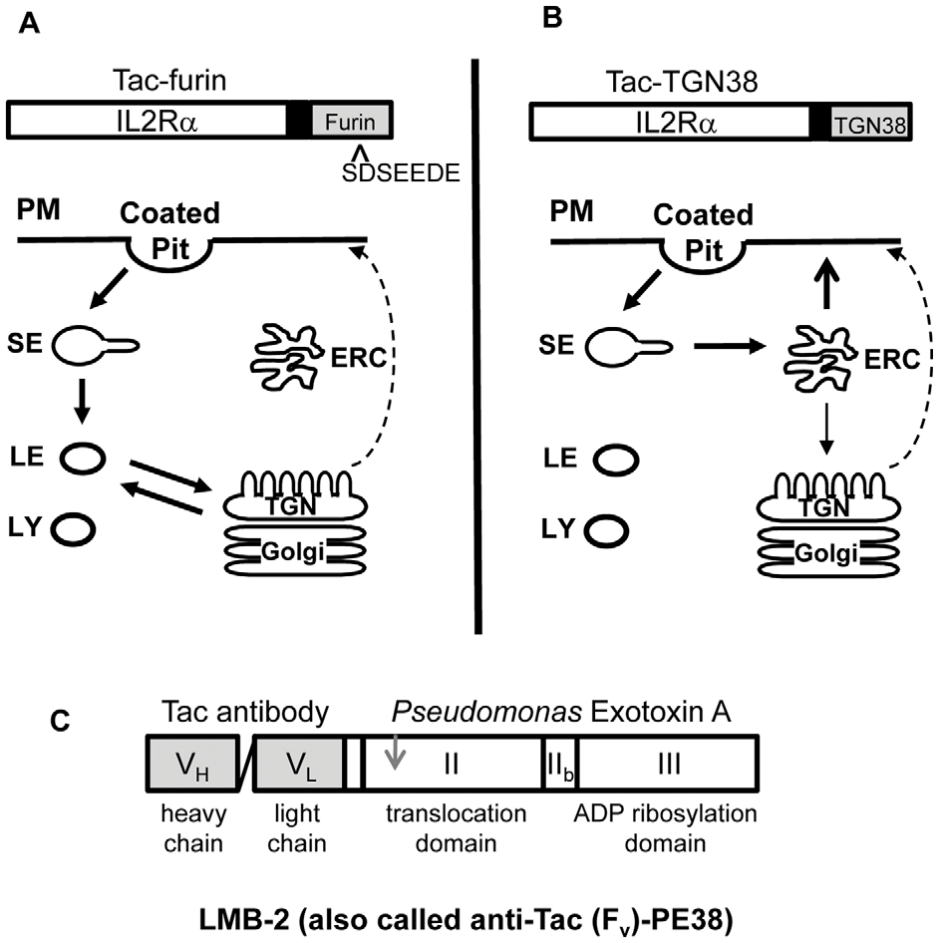
Transport pathways taken by Tac chimeras. Tac-furin (**A**) and Tac-TGN38 (**B**) are internalized from the plasma membrane (PM) and transported to the sorting endosome (SE). From here, Tac-TGN38 passes through the endocytic recycling compartment (ERC). Most of the Tac-TGN38 returns to the PM, but about 20% of the molecules in the endocytic recycling compartment are trafficked to the trans Golgi network (TGN). In contrast, Tac-furin passes through maturing endosomes on its way to the TGN without accumulating to any observable degree in the endocytic recycling compartment. Dashed line indicates anterograde transport from TGN to PM. Above each panel are diagrams of the Tac chimeras showing the Tac lumenal (white) and transmembrane (black) domains, and the cytoplasmic tail domain of furin or TGN38 (gray), respectively. **C**) Stick representation of LMB-2 indicating the various domains of the immunotoxin. The variable domain (heavy and light chain) of the Tac antibody (gray) are linked with a small peptide linker. The truncated version of *Pseudomonas* exotoxin A (white) consists of the translocation domain (II) and the enzymatic domain (III), but lacks the wild type binding domain. The gray arrow indicates an essential proteolytic processing site after amino acid 279.

Protein toxins have been developed as components of anti-cancer therapies due to their potent cell killing ability. Immunotoxins contain a cell-binding moiety based on an antibody that has specificity for tumor cell antigens attached to a portion of a plant or bacterial toxin. LMB-2 is an immunotoxin comprised of a truncated form of *Pseudomonas* exotoxin A (PE) fused to the variable region of an antibody that binds the Interleukin 2 Receptor (IL2R) α-chain (also known as anti-Tac antibody), which acts as the binding domain (Figure 1C) [1], [2]. The toxic PE fragment contains the processing, translocation and ADP ribosylation domains. The IL2R is present on a wide variety of hematologic malignancies and on normal T cells that mediate graft rejection and graft versus host disease, while resting T and B cells display little IL2R [3]. In preclinical trials, LMB-2 inhibited protein synthesis in IL2R+ transfected epidermoid carcinoma cells and caused complete tumor regression in tumor-bearing nude mice [4]. In clinical trials this immunotoxin was shown to be effective against some IL2R+ hematologic malignancies, including refractory hairy cell leukemia [5].

In order to achieve maximal killing efficiency, PE must be proteolytically processed and undergo retrograde transport to the endoplasmic reticulum. Wild type PE binds to LDL-Receptor Related Protein 1 (LRP1) and enters the cell by receptor-mediated endocytosis [6]. In some cell types, a portion of the toxin-receptor complex is associated with detergent-resistant membranes, though this is not required for efficient internalization of PE or subsequent cytotoxicity [7]. Following cell entry, the ligand-receptor complex undergoes retrograde transport through endosomes to the Golgi apparatus, a process that is dependent in part on the small GTPase Rab9, suggesting transit via late endosomes [7], [8]. The toxin ultimately reaches the endoplasmic reticulum using multiple transport pathways, including both Rab6- and Arf1-dependent steps as well as the KDEL mediated pathway. A C-terminal sequence (REDL) is critical for retrograde transport to the ER using the KDEL receptor retrieval system. During transport, PE is cleaved by the endopeptidase, furin, into a 28 kDa N-terminal fragment and a 37 kDa C-terminal fragment that contains ADP ribosylation activity [9], [10]. The fragments are joined by a disulfide linkage, which must be reduced for translocation of the 37 kDa fragment into the cytoplasm [11]. After translocation out of the ER and into the cytosol, PE catalyzes the ADP ribosylation of cellular elongation factor 2, leading to inhibition of protein synthesis and cell death [12], [13].

Several membrane proteins undergo transport through endosomes to the trans Golgi network. These include furin [14], [15], [16], [17], TGN38 [18], [19], [20], and the cation-independent mannose-6-phosphate receptor [21]. It has been found that these proteins actually use a variety of intracellular itineraries between the plasma membrane and the TGN. In previous studies, we examined the trafficking of chimeras consisting of an extracellular IL2R α-chain (Tac) domain linked to intracellular and transmembrane domains of furin or TGN38. Using fluorescently labeled anti-Tac antibodies, we followed the intracellular itineraries of these chimeras after endocytosis from the cell surface [22], [23], [24].

A model depicting the intracellular transport of these constructs is summarized in Figure 1 (A, B). As shown in previous publications [22], [23], [24], [25], both constructs are delivered initially to early sorting endosomes. The Tac-furin construct remains associated with the sorting endosomes as they begin to mature into late endosomes [24]. The Tac-furin leaves the late endosomes about 20–30 minutes after internalization, and it appears in the TGN at about the same time. Subsequently, the Tac-furin cycles between the TGN and late endosomes. The intracellular pool of Tac-furin returns to the cell surface with a t_1/2_ of about 30 minutes.

Tac-TGN38 goes from the sorting endosomes to the endocytic recycling compartment with kinetics indistinguishable from the recycling transferrin receptor [22]. On each passage through the cell, about 80% of the Tac-TGN38 returns to the cell surface, again with the same kinetics as transferrin receptors. The remaining Tac-TGN38 remains intracellular and is delivered to the TGN. The intracellular pool of Tac-TGN38 returns to the cell surface with a t_1/2_ of about 45 minutes.

Immunotoxin-induced cytotoxicity is dependent on retrograde delivery from the TGN to the endoplasmic reticulum, and processing during its delivery may enhance its potency. Generation of the proteolytically processed, more active immunotoxin may depend on the transport kinetics and specificity of movement through the various intracellular organelles. Both these constructs deliver Tac antibodies to the TGN, but the itineraries to get there are very different. In particular, the Tac-furin construct passes through maturing endosomes, so it would presumably be exposed to endogenous furin for the greatest time en route to the TGN. The Tac-TGN38 construct is never found in detectable amounts in late endosomes. From the TGN, a fraction of the anti-Tac-containing LMB-2 must move by retrograde transport through the Golgi apparatus and then to the ER. We have not been able to detect this retrograde transport beyond the TGN using fluorescence microscopy.

In this set of experiments, cells stably expressing Tac-TGN38 and Tac-furin were treated with LMB-2, and subsequent cell killing was assayed. Herein, we report significant differences in the cytotoxicity of LMB-2, depending on which chimeric construct brings it into the cell. LMB-2 that is targeted through the endocytic recycling compartment to the TGN via Tac-TGN38 kills cells more efficiently than LMB-2 delivered through the late endosomes by Tac-furin. We also found that LMB-2 is degraded more rapidly in cells expressing Tac-furin than in Tac-TGN38 cells. Collectively these data provide evidence that endocytic trafficking of immunotoxins significantly affects their cytotoxic potency.

## Experimental Methods

### Materials

Ham's F12 medium, penicillin/streptomycin and fetal bovine serum were from Invitrogen (Carlsbad, CA). Hygromycin B was from Calbiochem (LaJolla, CA). G418 sulfate was from Mediatech (Herndon, VA). Alexa546 protein labeling kit and goat anti-mouse-Alexa 546 were from Molecular Probes (Eugene, OR). Bovine serum albumin, TritonX-100, saponin, Hoechst 33258, anti-*Pseudomonas* exotoxin A polyclonal antibody, anti-tubulin monoclonal antibody (clone DM1A) and other standard chemicals were from Sigma Chemical (St Louis, MO). Monoclonal antibodies against Tac were purified from ascites fluid prepared from the hybridoma cell line 2A3A1H (ATCC, Manassas, VA) using protein G affinity chromatography (GE Life Sciences, Piscataway, NJ). Bicinchoninic assay, Super Signal chemiluminescence substrate, and horseradish peroxidase (HRP)-conjugated antibodies were from Pierce/Thermo-Fisher (Rockford, IL). Odyssey blocking buffer, goat anti rabbit IRdye 800CW, and goat anti mouse IRdye 680 were from Li-Cor Biosciences (Lincoln, NE). Immobilon P membrane was from Millipore (Billerica, MA). Longlife SDS-Hepes acrylamide gels were from NuSep, Ltd. (Lawrenceville, GA).

### Cell culture

Tac-TGN38 and Tac-furin cells have been previously described [22], [24], [25]. Both are CHO cell lines that stably express the human transferrin receptor and the Tac chimeras. Cells were grown in bicarbonate-buffered Ham's F12 medium supplemented with 5% (v/v) fetal bovine serum, 2 g/L D-glucose, 0.05 units/L penicillin, 50 mg/L streptomycin, 200 mg/L G418 and 0.2 units/L hygromycin B and maintained in a humidified 37°C chamber with 5% CO_2_. Cells for multiwell plate reader spectroscopy were plated in 96 well plastic cell culture plates using a Multidrop automated dispenser (Titertek Instruments Inc, Huntsville, AL). Cells for microscopy experiments were plated in 96 or 384 well optical bottom culture plates using the Multidrop automated dispenser.

### Immunotoxin treatment

Cells were seeded in culture plates on day 1. On day 2, they were treated with LMB-2 or negative control toxin Erb38, an immunotoxin that binds to Erb2/HER2, which is not expressed in CHO cells. On day 3, the cells were assayed by several different methods to assess cytotoxicity, expression of the Tac chimeras and LMB-2 processing.

### Treatment with lysosomal protease inhibitors

Cells were seeded in culture plates on day 1. Cells were pretreated for 1 or 24 hours with 100 µM Leupeptin, E-64, or Chymostatin (all from Sigma Chemical Co.) prior to addition of LMB-2. For the 24 hours pretreatment cells were allowed to adhere to the dish for 2 hours before addition of the inhibitors. On day 2, they were treated with LMB-2. On day 3, the cells were assayed for cytotoxicity.

### Cell count using Hoechst dye

Cells were washed with PBS (137 mM sodium chloride, 2.7 mM potassium chloride, 10 mM dibasic sodium phosphate, 1.5 mM monobasic sodium phosphate, pH 7.4) using a Bio-Tek ELx405 Select Plate Washer. Dead cells are washed away during the rinses. Cells were fixed at room temperature for 10 minutes with 1.5% paraformaldehyde in PBS and then stained with 5 µg/ml Hoechst 33258 dye for 20 minutes at room temperature. Cells were imaged with a 10x dry objective, and the cell count for each condition was obtained by scoring Hoechst positive nuclei.

### Lactate dehyrogenase assay

Cells were washed 3 times with PBS, lysed with 2% TritonX-100 in PBS, and then a fraction of the lysate was used as the substrate in the Lactate dehyrogenase (LDH) assay (Cytotoxicity Detection Kit^Plus^, Roche Applied Biosciences). The lysates were incubated for 30 minutes with the assay reagents and the absorbance at 490 nm was read using a Spectromax spectrophotometer (Molecular Devices).

### Data analysis for EC_50_ measurement

Based on NIH recommendation for determination of EC_50_ in multi-well assays, a four parameter logistic model also called Hill slope model was used [26], [27], [28], [38].
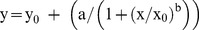
(1)Where, b = slope, y = concentration, 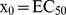 and a = maximum response

### Analysis of Tac expression by indirect immunofluorescence

Cells were fixed as described above, permeabilized with 0.025% saponin in PBS containing 0.2% bovine serum albumin, and then stained sequentially with 10 µg/ml anti-Tac antibody and goat anti-mouse IgG-Alexa546. Cells were subsequently stained with Hoechst dye as described above and then imaged.

### Microscopy

An ImageXpress^MICRO^ imaging system (Molecular Devices, Sunnyvale, CA) equipped with a 300W Xenon-arc lamp (Perkin-Elmer, Waltham, MA), 10x Plan Fluor 0.3 NA objective (Nikon, Inc. Melville, NY), and a Photometrics CoolSnapHQ camera (Roper Scientific, Tucson, AZ) was used to acquire images. Hoechst images were acquired using 377/50 nm excitation and 447/60 nm emission filters with a 415 dichroic long-pass filter. Alexa546 images were acquired using 543/22 nm excitation and 593/40 nm emission filters with a 569 dichroic long-pass filter. Images were acquired using 2×2 binning. Each site was individually focused using a high-speed laser autofocus comprised of a 690 nm diode laser and a dedicated 8-bit CMOS camera. 696×520 pixel images were acquired at 12 intensity bits per pixel.

### Image processing

Images were processed using Metamorph image analysis software (Molecular Devices, Downingtown, PA). All images used for fluorescence intensity measurements were corrected for shading artifacts and background fluorescence [29] before obtaining the total fluorescence intensity per field. Cell count was measured using the Cell Scoring application in Metamorph. Size and intensity over local background parameters were set to automatically determine the number of Hoechst-positive bright spots (i.e., nuclei) per field. The Tac fluorescence intensity per cell was calculated by dividing the total fluorescence intensity per field by the number of cells in the field.

### Analysis of LMB-2 degradation and processing by immunoblot

Cells were incubated with LMB-2, washed with PBS and lysed directly in 50 mM Tris pH 7.5, 150 mM NaCl, 5 mM EDTA, 1% SDS, pepstatin, leupeptin, aprotinin and AEBSF. DNA was sheared and the lysates were cleared by centrifugation at 14,000×g. Protein was quantified using the BCA assay. Protein samples were loaded on a 10% SDS acrylamide gel. Depending on the detection method blots were processed as follows: For imaging using Li-Cor system, proteins were blotted to nitrocellulose membrane and blocked in Odyssey blocking buffer. The membrane was incubated sequentially with anti-*Pseudomonas* exotoxin A antibody (1∶5000) and anti-tubulin antibody (1∶10000) as a loading control. Following primary antibody incubation, membranes were probed with secondary antibodies conjugated to IRdye 680 or 800 and imaged using a Li-Cor Odyssey system. To quantify the amount of processed LMB-2, the integrated intensity for full length and cleaved LMB-2 bands were background corrected using an average local correction value. The fraction of cleaved LMB-2 was calculated using the integrated intensity values for both bands. For detection by enhanced chemiluminescence, proteins were blotted to immobilon-P membrane, blocked with 10% milk in PBS-T, incubated with primary antibody as described above and subsequently with secondary antibodies conjugated to HRP.

## Results

### Cell killing by LMB-2 is more efficient in cells expressing Tac-TGN38 than in cells expressing Tac-furin

**Figure 2:**
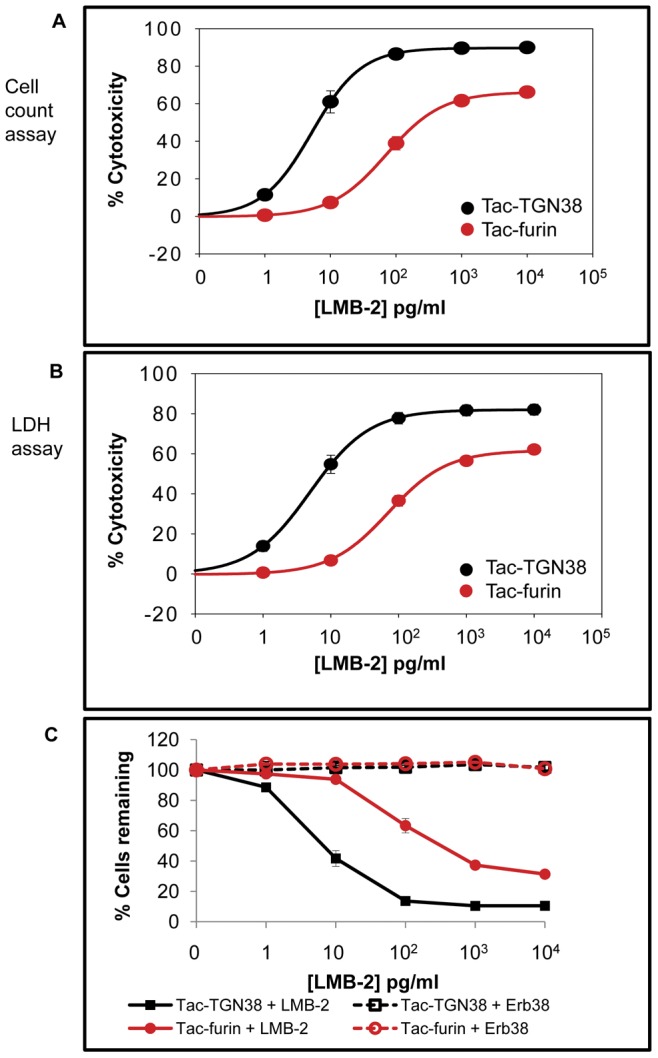
Comparison of cytotoxic effects and immunotoxin specificity in Tac-receptor expressing cells. Tac-TGN38 and Tac-furin cells were treated with up to 10 ng/ml of LMB-2. After a 24 hour incubation with immunotoxin, cells were washed and the cells remaining in the well were used for **A**) Hoechst-based cell count assay or **B**) LDH assay as described in Methods. Panels A and B show % cytotoxicity plotted against the concentration of LMB-2. The data points represent the cytotoxicity values averaged over several experiments, cell count assay (n = 9), LDH assay (n = 8). The standard error of the mean is shown. **C**) Tac-TGN38 and Tac-furin cells were seeded and then treated with up to 10 ng/ml of LMB-2 or Erb38 one day after plating. After a 24 hour incubation with immunotoxin, cytotoxicity was assessed by a cell count assay. The number of cells remaining after toxin treatment is shown as a fraction of cells incubated without toxin (n = 5). The standard error of the mean is shown.

To determine the effects of alternate transport routes on the LMB-2 killing efficiency, we compared cell killing by LMB-2 in cells expressing two Tac-chimeras that are delivered to the TGN by different routes (Figure 1). First, we examined how the induction of LMB-2 cytotoxicity varies with dose in the Tac-chimera expressing cell lines. Tac-TGN38 and Tac-furin cells were treated with 1 pg/ml to 10 ng/ml of LMB-2 for 24 hours. After the incubation with LMB-2, the percentage of remaining cells was determined using cell count and LDH assays (Figure 2A–B and Table 1). Both cell lines were killed by LMB-2 in a dose-dependent manner. We achieved about 90% killing of Tac-TGN38 cells at doses of LMB-2 above 0.1 ng/ml. Killing of the Tac-furin cells leveled off at about 65–80% at high concentrations. Even with 1000 ng/ml LMB-2 treatment, only 70% killing was observed in Tac-furin cells (data not shown). The observed cytotoxicity was specific for LMB-2 since cell lines incubated with Erb38 [30], an immunotoxin that binds to Erb2/HER2, which is not expressed in CHO cells, remained viable (Figure 2C). These data indicate that the Tac-TGN38 cells are more efficiently killed by LMB-2 than the Tac-furin cells.

**Table 1:** The data in Figure 2 were fit in SigmaPlot using a four parameter logistic model (Hill-Slope model) with the equation y = y_0_+(a/(1+(x/x_0_)^b^)) where b = slope, y = concentration, x_0_ = EC_50_ and a = maximum response [26], [27], [28], [38].

	Cell count assay		LDH assay	
Cell line	Maximum killing (%)	EC_50_ (pg/ml)	Maximum killing (%)	EC_50_ (pg/ml)
Tac-TGN38	90+/−2	6+/−1	82+/−3	6+/−1
Tac-furin	67+/−2	83+/−17	62+/−2	75+/−14

The p-value for EC_50_ when calculated for Cell count assay was 0.0003, and for LDH assay was <0.0001. The p-values for Maximum % killing were <0.0001 for both cell count and LDH assays.

The killing effectiveness of the LMB-2 toxins based on the concentration required to achieve half-maximal killing was compared in these cell lines. As shown in Table 1, half-maximal killing (EC_50_) was achieved in Tac-TGN38 cells at concentrations approximately 15-fold lower than the concentration needed for half-maximal killing of Tac-furin cells.

### Selective resistance to LMB-2 in cells expressing lower levels of Tac-furin

**Figure 3:**
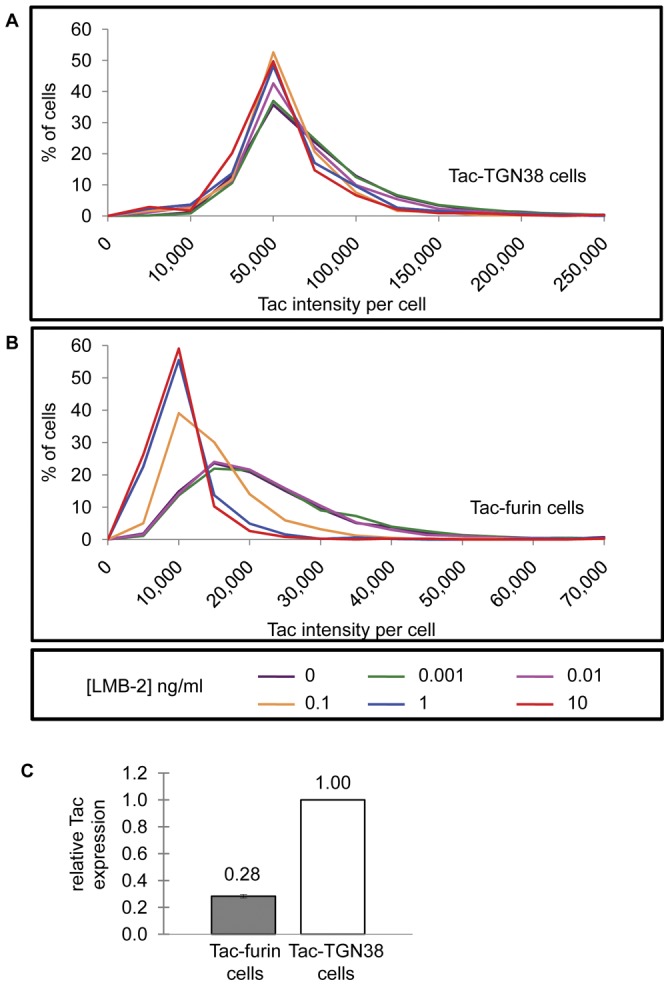
Effects of heterogeneous Tac expression on LMB-2 cytotoxicity. Tac-TGN38 (**A**) and Tac-furin (**B**) cells were treated for 24 hours with doses of LMB-2 up to 10 ng/ml. Cells were then fixed, permeabilized and stained with anti-Tac monoclonal antibody and Alexa546 conjugated secondary antibody. The histograms show the frequency distribution of Tac intensity for each cell line after exposure to varying concentrations of LMB-2. The experiments were replicated at least 3 times for each cell line, and representative data from one experiment are shown. **C**) Tac-TGN38 and Tac-furin cells were fixed and then stained with Hoechst dye, anti-Tac monoclonal antibody and subsequently with Alexa546 conjugated secondary antibody. The average fluorescence per cell was measured as described in Methods. Images were subjected to background and shade correction before intensity calculations. Fluorescence intensity per field was divided by the cell number to calculate the average intensity per cell. The data are the average of 9 experiments. The standard error of the mean is shown.

We noted that there were some surviving cells even after 24 hours at the highest concentration of the immunotoxin, and the percentage of survivors was higher for the Tac-furin cells than for the Tac-TGN38 cells (Figure 2). The resistant cells, particularly in the Tac-furin cell line, were due to heterogeneity in expression of the constructs. Attempts were made to reclone these cell lines, but heterogeneity of expression returned within a few passages of the cells. Thus, we tested whether LMB-2 preferentially kills cells with higher levels of Tac expression within the cell population since this might account for differences in sensitivity to the immunotoxins. Tac expression was quantified by immunofluorescence microscopy in cells that survived a 24 hour incubation with the immunotoxin at different concentrations. Cells were treated with LMB-2 for 24 hours, fixed and stained with anti-Tac antibody and a secondary antibody tagged with Alexa546. The fluorescence intensity per cell was determined (Figure 3A–B). Both cell lines exhibit heterogeneous expression of Tac as indicated by the broad intensity distribution in untreated cells (Figure 3A–B, purple lines; 0 ng/ml). A shift towards lower levels of Tac expression was observed in surviving Tac-furin cells, particularly with doses of LMB-2 greater than 0.1 ng/ml. These data suggest that LMB-2 preferentially kills cells that express high levels of the Tac epitope relative to other lower expressing cells in the same pool.

**Figure 4:**
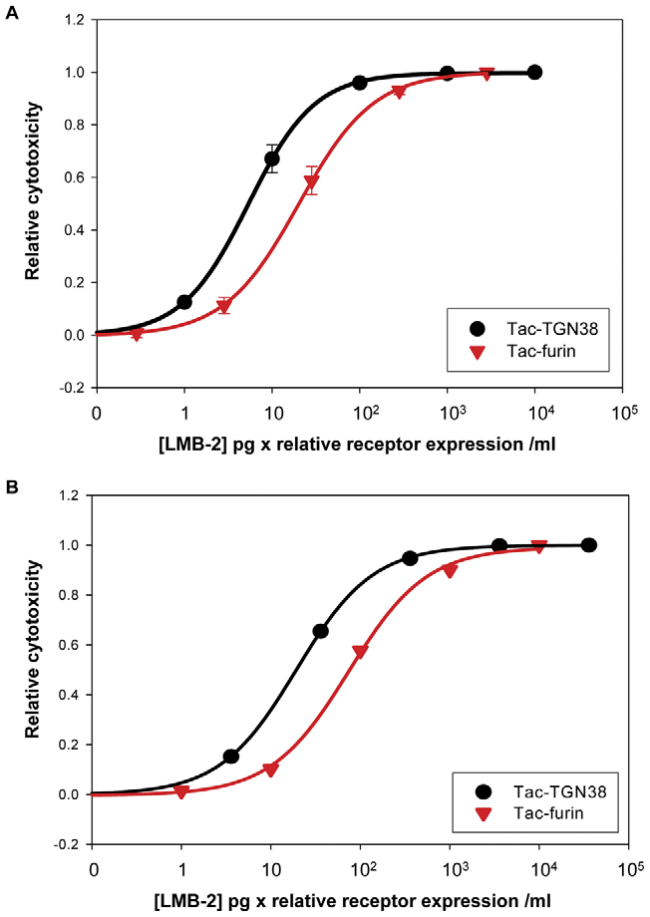
Comparison of cell toxicity at comparable levels of immunotoxin binding. Tac-TGN38 and Tac-furin cells were treated with the indicated dose of LMB-2. After a 24 hour incubation with immunotoxin, cells were washed, and the cells remaining in the well were used for **A**) Hoechst-based cell count assay or **B**) LDH assay as described in Methods. Panels A and B show the relative cytotoxicity plotted against the amount of LMB-2 adjusted to reflect the different Tac expression levels (Figure 3C) in the cell lines ([LMB-2] × Tac expression). Here the cytotoxicity has been normalized so that a value of 1 represents the maximum killing observed with the highest dose of LMB-2 for each cell line (relative cytotoxicity). The data points represent the cytotoxicity values averaged over several experiments, cell count assay (n = 9), LDH assay (n = 8). The standard error of the mean is shown.

Tac-furin is expressed at about one-third the level of Tac-TGN38 in the cell lines (Figure 3C). Thus, we determined cell toxicity at comparable levels of toxin binding to the two cell lines. We observed that saturation of anti-Tac binding was achieved at concentrations at or above 1000 ng/ml (data not shown), so the concentrations used for cell killing are in the nearly linear part of the binding curve. The product of the concentration of the LMB-2 and the relative Tac-expression per cell should then give a value proportional to the amount of bound toxin for each cell line. The relative cytotoxicity is plotted vs. the relative toxin binding in Figure 4. The relative EC_50_'s calculated based on Tac expression for cell count assay were 6 and 24 pg × relative receptor expression/ml for Tac-TGN38 and Tac-furin, respectively (Table 2). The cells expressing Tac-TGN38 are about 4-fold more sensitive to the bound toxin.

**Figure 5:**
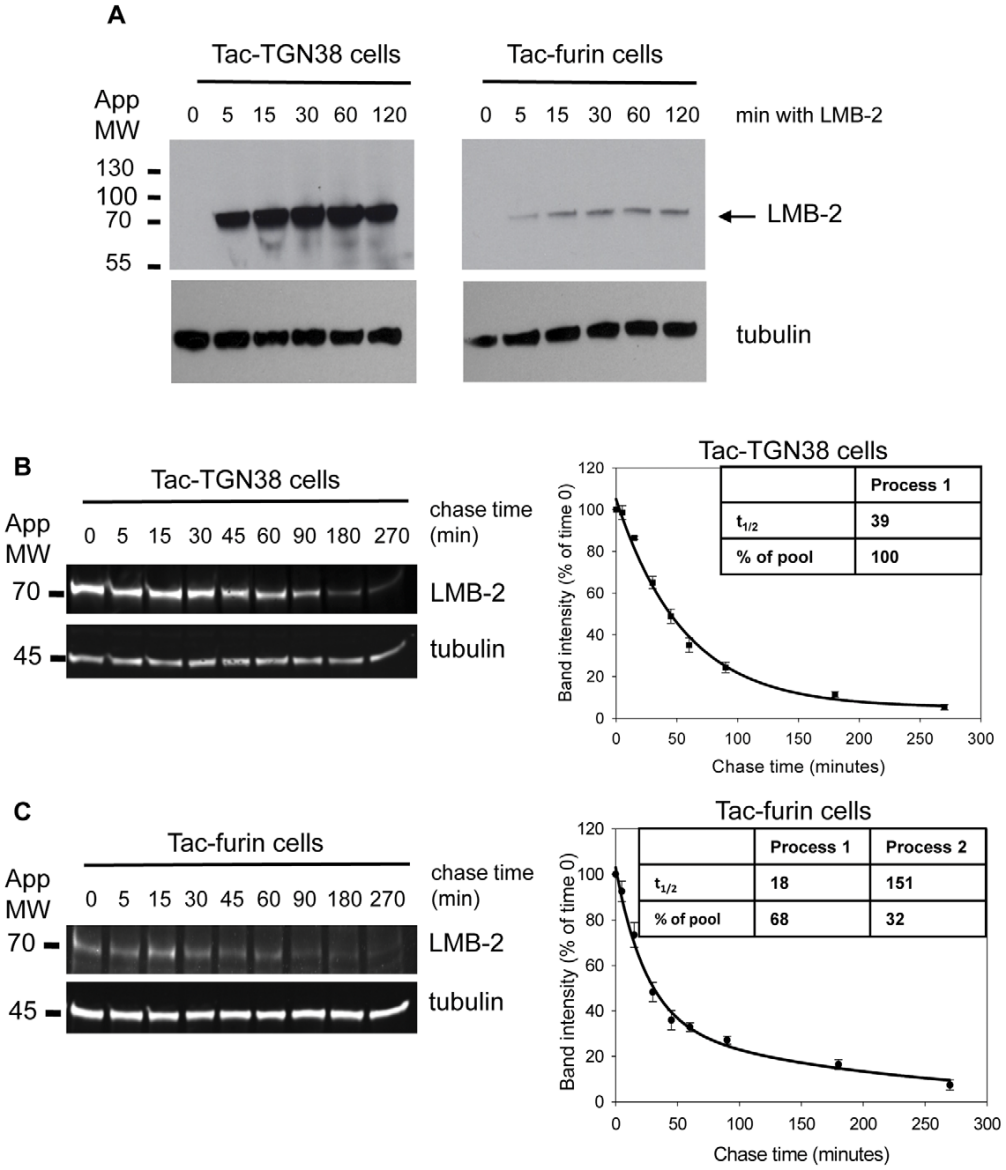
Analysis of LMB-2 processing and degradation. **A**) Tac-TGN38 and Tac-furin cells were seeded, and one day after plating the cells were incubated with 1 µg/ml of LMB-2 for 5, 15, 30, 60 or 120 minutes or left untreated. At each time point cells were lysed and proteins were analyzed by immunoblot with anti-*Pseudomonas* exotoxin A antibody and anti-tubulin antibodies. Proteins were detected using enhanced chemiluminescence reagents. This experiment was replicated 2 times with similar results, and one set of representative blots are shown. Tac-TGN38 (**B**) and Tac-furin (**C**) cells were incubated for 5 minutes with 750 ng/ml LMB-2 and then chased in immunotoxin-free medium for up to 270 minutes. At each time point cells were lysed in SDS. Samples were adjusted so that the amount of full length LMB-2 was the same at time 0 for each cell line. Subsequent analysis was by immunoblot using an antibody for *Pseudomonas* exotoxin A. LMB-2, tubulin and molecular weight standards are indicated. Individual bands were quantified using Metamorph. The right panels show the normalized band intensities at different chase times averaged over 4 experiments +/− standard error of the mean. The kinetic data for Tac-TGN38 were fit using SigmaPlot based on a single three parameter equation for exponential decay y  =  y_0_ + ae^−bt^, and Tac-furin data were fit using four parameter equation for exponential decay with the equation y  =  ae^−bt^+ce^−dt^ (where a+c  = 100). Graph insets show the calculated t_1/2_ values for each cell line.

**Table 2:** t_1/2_ for degradation were based on exponential decay curve fitting equation (see figure 5 legend for equation).

Cell line	t_1/2_ for degradation	Corrected EC50 Cell count assay (pg × relative receptor expression/ml)
Tac-TGN38	39 min	6+/−1
Tac-furin	18 min (68%) and 151 min (32%)	24+/−5

The EC_50_'s were also calculated after correcting for Tac expression and fitted in SigmaPlot using a four parameter logistic model (Hill-Slope model) with the equation y = y_0_+(a/(1+(x/x_0_)^b^)) where b = slope, y = concentration, x_0_ = EC_50_ and a = maximum response. [26], [27], [28], [38]. The p-values for Tac-TGN38: Tac-furin were 0.03.

### The turnover rate of LMB-2 is significantly faster when LMB-2 traffics to the TGN via the late endosomal pathway

After endocytosis, a large fraction of the internalized toxin is degraded. In the continued presence of LMB-2 in the medium, the amount of LMB-2 in cells reached a steady level within 30–60 minutes (Figure 5A), indicating that continued uptake was balanced by degradation. We measured the rate of LMB-2 degradation in cells incubated with 750 ng/ml LMB-2 for 5 minutes followed by a chase period of up to 270 minutes. Cells were harvested, and LMB-2 levels were assessed by immunoblot using anti-*Pseudomonas* exotoxin A antibody (Figure 5 B, C). The t_1/2_ of LMB-2 degradation in Tac-TGN38 cells was 39 min. In Tac-furin cells two distinct rates were observed with t_1/2_ values of 18 min and 151 min (Figure 5 B, C graph inserts). Since the majority of the Tac-furin was degraded by the faster process, the overall t_1/2_ of degradation was faster for Tac-furin than for Tac-TGN38. This shows that the endocytic pathway taken by the immunotoxin contributes significantly to its lifetime in cells.

**Figure 6:**
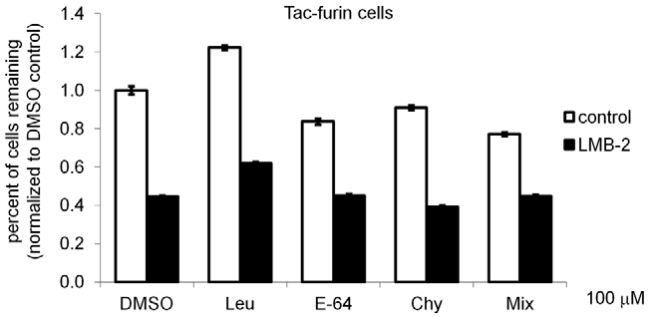
Effect of lysosomal protease inhibitors on LMB-2 cytotoxicity. Tac-furin cells were pretreated with 100 µM Leupeptin (Leu), E-64, Chymostatin (Chy), a combination of all three inhibitors or DMSO control for 24 hours. Cells were subsequently treated with 1 ng/ml LMB-2 in the continued presence of inhibitors. After a 24 hour incubation with immunotoxin, cells were counted using the Hoechst-based assay. The number of cells remaining after toxin treatment is shown as a fraction of cells incubated without toxin normalized to the DMSO control (n = 6 wells). The standard error of the mean is shown. Similar experiments were performed twice and one representative data set is shown.

Since much of the LMB-2 was being degraded quickly in Tac-furin cells, we tested whether lysosomal protease inhibitors, which could block LMB-2 degradation in the lysosome, would increase the toxicity of LMB-2 in these cells. Cells were treated with leupeptin, E-64, chymostatin, or a combination of inhibitors for 1 hr (not shown) or 24 hours. Then cells were incubated with LMB-2 for 24 hours in the continued presence of inhibitors and assayed for toxicity using the Hoechst-based cell count assay. No significant difference in cell toxicity was observed in any condition we tested compared to the solvent control (Figure 6). Under the same conditions, the lysosomal degradation of internalized α2-macroglobulin was reduced significantly (data not shown), verifying that lysosomal proteolysis was reduced.

### LMB-2 cleavage to the 37 kDa form depends on specific intracellular transport pathways

Selective proteolytic processing is required for efficient immunotoxin-induced killing [9], [10]. To assess whether LMB-2 was processed differently in the various Tac expressing cell lines, we examined the cleavage of full-length LMB-2 to produce the 37 kDa C-terminal fragment that contains ADP ribosylation activity. Cells were incubated for 180 minutes with 1000 ng/ml LMB-2, after which cells were lysed in SDS and analyzed by immunoblot using an anti-*Pseudomonas* exotoxin A antibody (data not shown). To quantify which cells have more efficient cleavage we compared the amount of full length LMB-2 to cleaved LMB-2 in each cell line. We noted that Tac-furin cells have a higher ratio of cleaved to uncleaved compared to TacTGN38 cells (11% and 3%, respectively). These data suggest that efficiency of LMB-2 processing depends on intracellular transport pathways.

## Discussion

Wild type *Pseudomonas* exotoxin A follows a complex trafficking pattern using multiple pathways [7] and undergoes processing in endosomal compartments. Understanding the effects of different pathways the toxin could utilize to traffic through the cell would be advantageous for development of more potent immunotoxins for targeting cancer cells. Several exotoxin-derived chimeric toxins (e.g., TGFα-PE38, [31]) require proteolytic cleavage for maximal cytotoxic activity. For wild type PE this cleavage occurs between amino acids 279 and 280, and it is likely mediated by furin [9], [10]. PE-based immunotoxins engineered to contain only amino acids 280–613 are more toxic than those containing the cleavage site [32]. For LMB-2 the proteolytic processing produces a cytotoxic 37 kDa C-terminal fragment.

Immunotoxins utilize a variety of cell-specific binding moieties to gain entry into the cell, thus the comparative effects of different trafficking pathways are difficult to evaluate. Moreover, wild type PE can take advantage of both lipid and protein based trafficking routes following internalization by its receptor [7]. We examined LMB-2 induced cytotoxicity in cell lines that use the same extracellular epitope for binding the immunotoxin but reach the TGN using different itineraries. We used previously characterized Tac-expressing cell lines to identify how utilizing various transport pathways can affect LMB-2 toxicity. LMB-2 was targeted through either the recycling pathway or the late endosome pathway to reach the TGN (Figure 1).

We found that LMB-2 is most effective when targeted through the endocytic recycling compartment using Tac-TGN38, and it is less effective when it is targeted through the late endosome pathway by Tac-furin. LMB-2 bound to Tac-furin would spend the most time in compartments containing the activating protease furin, and this route of delivery does lead to the highest fraction of the activated 37 kDa fragment. However, LMB-2 internalized via Tac-furin is degraded faster than LMB-2 internalized via Tac-TGN38. The faster degradation in Tac-furin expressing cells is presumably due to exposure to lysosomal proteases in maturing endosomes before the LMB-2 is sorted to the TGN. LMB-2 internalized by Tac-TGN38 avoids this exposure to proteolytically active endosomes after internalization. Additionally, some Tac-furin is sorted to proteolytically active late endosomes after passage through the TGN. Thus, even though LMB-2 internalized via Tac-furin yields a higher fraction of the activated 37 kDa fragment, it is less effective in cell killing than LMB-2 internalized via Tac-TGN38.

Nearly all of the LMB-2 that is internalized by either Tac-TGN38 or Tac-furin is degraded within 5 hours (Figure 5), so cell killing is brought about by a small fraction of the total internalized immunotoxin. The most effective routes for cytotoxicity may be those that increase the avoidance of proteolysis in late endosomes and lysosomes. Indeed, it has been shown that a PE-based immunotoxin that has been modified to remove several lysosomal cleavage sites is highly effective at killing cells [33]. We attempted to increase the efficiency of killing by LMB-2 in Tac-furin cells by inhibiting lysosomal proteases, but no enhancement was observed (Fig. 6).

Development of the most efficient and effective immunotoxin should consider all the possible transport and sorting steps. This would include maximizing exposure to activating proteolysis by furin, minimizing exposure to general protein degradation in late endosmes, and efficient delivery to the TGN and transport back to the ER. These parameters will be influenced by the choice of the binding target for the antibody portion of the immunotoxin.

The relevant pathways for LMB-2 transport would be those utilized by IL2R, the endogenous receptor for LMB-2. The IL2 receptor is comprised of three subunits, α, β and γ, which undergo a complex trafficking pattern following endocytosis [34]. The αβγ trimer constitutes the high affinity IL-2 receptor, and associates with detergent-resistant membranes upon ligand binding [35], [36]. The α subunit, which is the Tac antibody binding domain [4], dissociates from the other subunits in early sorting endosomes and recycling endosomes, and it is predominantly recycled to the plasma membrane. This is most similar to trafficking of LMB-2 by TacTGN38 in the present study, which suggests that it would be an effective route of entry for cell killing. The β and γ subunits are sorted towards the degradation pathway, so immunotoxins targeting these subunits would be expected to be less effective since they would presumably be degraded rapidly. Indeed, a similar immunotoxin targeting the β subunit of the IL-2 receptor was cytotoxic to cells expressing the β subunit of the IL-2 receptor, but generally higher concentrations were needed than for an immunotoxin targeting the α subunit [37]. Direct comparisons may be difficult, however, because of differences in subunit expression levels and antibody affinities.

In cancer cells treated with LMB-2, it is not clear in which organelle the immunotoxin dissociates from the IL2R, or precisely how it is transported back to the ER. Further studies to examine the trafficking and processing of LMB-2 treated cancer cells are needed to address this issue.
